# Unveiling *DENND2D* as a Novel Prognostic Biomarker for Prostate Cancer Recurrence: From Gene to Prognosis

**DOI:** 10.3390/biomedicines13010025

**Published:** 2024-12-26

**Authors:** Chi-Fen Chang, Lih-Chyang Chen, Yei-Tsung Chen, Chao-Yuan Huang, Chia-Cheng Yu, Victor C. Lin, Te-Ling Lu, Shu-Pin Huang, Bo-Ying Bao

**Affiliations:** 1 Department of Anatomy, School of Medicine, China Medical University, Taichung 406, Taiwan; cfchang@mail.cmu.edu.tw; 2 Department of Medicine, Mackay Medical College, New Taipei City 252, Taiwan; lihchyang@mmc.edu.tw; 3 Department of Life Sciences and Institute of Genome Sciences, National Yang Ming Chiao Tung University, Taipei 112, Taiwan; yeitsungchen@ym.edu.tw; 4 Department of Urology, National Taiwan University Hospital, College of Medicine, National Taiwan University, Taipei 100, Taiwan; cyh0909@ntu.edu.tw; 5 Division of Urology, Department of Surgery, Kaohsiung Veterans General Hospital, Kaohsiung 813, Taiwan; ccyu@vghks.gov.tw; 6 Department of Urology, School of Medicine, National Yang Ming Chiao Tung University, Taipei 112, Taiwan; 7 Department of Pharmacy, College of Pharmacy and Health Care, Tajen University, Pingtung 907, Taiwan; 8 Department of Urology, E-Da Hospital, Kaohsiung 824, Taiwan; ed102161@edah.org.tw; 9 School of Medicine for International Students, I-Shou University, Kaohsiung 840, Taiwan; 10 Department of Pharmacy, China Medical University, Taichung 404, Taiwan; lutl@mail.cmu.edu.tw; 11 Graduate Institute of Clinical Medicine, College of Medicine, Kaohsiung Medical University, Kaohsiung 807, Taiwan; 12 Department of Urology, Kaohsiung Medical University Hospital, Kaohsiung 807, Taiwan; 13 Institute of Medical Science and Technology, College of Medicine, National Sun Yat-Sen University, Kaohsiung 804, Taiwan

**Keywords:** prostate cancer, recurrence, *DENN* domain-containing genes, gene set enrichment analysis, biomarker

## Abstract

**Background:** Prostate cancer is a major global health burden, with biochemical recurrence (BCR) following radical prostatectomy affecting 20–40% of patients and posing significant challenges to prognosis and treatment. Emerging evidence suggests a critical role for differentially expressed in normal and neoplastic cell (*DENN*) domain-containing genes in oncogenesis; however, their implications in prostate cancer and BCR risk remain underexplored. **Methods:** This study systematically evaluated 151 single-nucleotide polymorphisms in *DENN* domain-containing genes in 458 patients with prostate cancer and BCR, followed by validation in an independent cohort of 185 patients. **Results:** Multivariate Cox regression analyses identified *DENND2D* rs610261 G>A as significantly associated with improved BCR-free survival in both cohorts (adjusted hazard ratio = 0.39, 95% confidence interval = 0.23–0.66, *p* = 0.001). Functional analysis revealed rs610261’s regulatory potential, with the protective A allele correlating with increased *DENND2D* expression in various human tissues. Compared to normal prostate tissues, *DENND2D* expression was reduced in prostate cancer, with higher expression being linked to favorable patient prognosis (*p* = 0.03). Gene set enrichment analysis revealed an association between *DENND2D* expression and the negative regulation of *MYC* target genes, including *MAD2L1*, *ERH*, and *CLNS1A*, which are overexpressed in prostate cancer and associated with poor survival. Furthermore, the elevated *DENND2D* expression promotes immune infiltration in prostate cancer, supporting its role in immune modulation. **Conclusions:** *DENND2D* is a prognostic biomarker for BCR in prostate cancer and offers new avenues for personalized treatment strategies.

## 1. Introduction

Prostate cancer is the second most common cancer among men worldwide, with significant morbidity and mortality rates [1]. Radical prostatectomy (RP) is the primary treatment for localized prostate cancer. However, a substantial number of patients, ranging from 20% to 40%, experienced biochemical recurrence (BCR) characterized by an increase in serum prostate-specific antigen (PSA) levels and signaling potential disease progression [2]. Despite advancements in imaging and clinical staging, predicting which patients are at risk for BCR remains challenging. Current prognostic tools depend on preoperative and pathological factors, such as the Gleason score, tumor stage, and surgical margins. However, these metrics often fail to account for molecular heterogeneity, which limits their predictive accuracy [3]. Various molecular biomarkers, such as gene expression profiles and epigenetic markers, have shown potential to supplement clinical parameters and improve prediction accuracy [4,5,6]. Conversely, their applicability is limited owing to the variability in outcomes and lack of validation across diverse populations. Identifying novel biomarkers associated with BCR transformed the clinical decision-making process and offered a pathway for personalized therapy in patients with prostate cancer.

Differentially expressed in normal and neoplastic cells (*DENN*) domain-containing genes serve as guanine nucleotide exchange factors for Rab GTPases. Genes containing the *DENN* domain facilitate the activation of Rab by replacing GDP with GTP, as activated Rabs are instrumental in various cellular processes, including vesicle trafficking, autophagy, and cellular signaling—which are crucial for maintaining cellular homeostasis and regulating immune responses [7]. Recent studies have suggested that the dysregulation of *DENN* domain-containing genes may be implicated in oncogenesis, including cancer proliferation, invasion, and metastasis. Alterations in *DENN* domain-containing genes, such as loss-of-function mutations, may contribute to cancer pathogenesis through the dysregulation of cellular signaling pathways, apoptosis, and autophagy [8]. *DENN* splice variants modulate tumor necrosis factor-mediated apoptosis, and are associated with resistance to chemotherapeutic agents [9]. *DENND2D* is a potential tumor suppressor in gastric cancer, and its downregulation correlates with poor prognosis and early recurrence [10]. Recently, *DENN* domain-containing gene variants were determined to be involved in multiple cancers, including breast and pancreatic cancer, suggesting their involvement in tumorigenesis and disease prognosis [11,12]. However, *DENN* domain-containing gene variants’ involvement in prostate cancer, specifically in BCR prediction, is largely unexplored.

Considering the emerging significance of *DENN* domain-containing genes in cancer treatment, we hypothesized that the genetic variants of these genes may be associated with the risk of BCR post-RP in patients with prostate cancer. We systematically evaluated 151 single-nucleotide polymorphisms (SNPs) across various *DENN* domain-containing genes in a discovery cohort comprising 458 patients with prostate cancer who underwent BCR. We then validated our data using an independent cohort of 185 patients and performed functional assays to elucidate the involvement of *DENND2D* in prostate cancer progression.

## 2. Materials and Methods

### 2.1. Study Population and Data Collection

Overall, 644 patients with histopathologically confirmed prostate cancer who underwent RP were recruited from three medical centers in Taiwan: National Taiwan University Hospital, E-Da Hospital, and Kaohsiung Medical University Hospital [5,13]. All participants were Han Taiwanese, had no genetic relationships, and did not receive adjuvant hormone or radiation therapy following RP. Clinical and demographic data, such as age at diagnosis, PSA levels, pathologic Gleason scores, cancer staging, surgical margin status, lymph node involvement, and BCR status, were collected from patient records. BCR was defined as two consecutive PSA levels of ≥0.2 ng/mL post-RP [14,15]. BCR-free survival time was calculated from the date of RP until BCR onset or last recorded follow-up. A two-phase study design was used to examine the effects of the genetic variants of the *DENN* family genes on patient outcomes. The discovery cohort, comprising 457 patients, was recruited from the National Taiwan University Hospital and E-Da Hospital, whereas the replication cohort, comprising 187 patients, was recruited from the Kaohsiung Medical University Hospital. Table S1 summarizes the baseline clinical and pathological characteristics of both cohorts, showing no significant differences in variables, such as age at diagnosis, PSA levels, pathological Gleason score, and lymph node metastasis between the cohorts. The PSA level at diagnosis, pathological Gleason score, and stage were significantly associated with BCR-free survival in both cohorts. The median follow-up times were 38 and 74 months with 137 (30.0%) and 92 (49.2%) BCR cases, for the discovery and replication cohorts, respectively. The study protocol was approved by the Institutional Review Board of Kaohsiung Medical University Hospital (KMUHIRB-2013132), and all participants provided written informed consent.

### 2.2. SNP Selection and Genotyping

Haplotype-tagging SNPs were selected from 17 genes within the *DENN* family (*DENND1A*, *DENND1B*, *DENND1C*, *DENND2A*, *DENND2B*, *DENND2C*, *DENND2D*, *DENND3*, *DENND4A*, *DENND4B*, *DENND4C*, *DENND5A*, *DENND5B*, *DENND6A*, *DENND6B*, *DENND10*, and *DENND11*) using Haploview v4.2. The criteria included a minor allele frequency (MAF) > 0.05 and pairwise linkage disequilibrium correlation coefficient (r^2^) exceeding 0.8, based on data from the 1000 Genomes Project for Han Chinese in Beijing and Southern Han Chinese populations [16,17].

Genomic DNA was extracted from 5 mL of whole blood collected in EDTA tubes using the QIAamp DNA Blood Maxi Kit (Qiagen, Valencia, CA, USA) according to the manufacturer’s instructions. Standardized protocols were applied across all centers, ensuring processing within 24 h of collection and storage at −80 °C prior to genotyping. Genotyping was conducted at Taiwan’s National Center for Genome Medicine using the Affymetrix Axiom Genotyping Array system (Thermo Fisher Scientific, Waltham, MA, USA) according to established methods [18]. SNPs with a genotyping call rate < 95%, MAF < 0.05, or deviation from the Hardy–Weinberg equilibrium (*p* < 0.001) were excluded; thus, 151 SNPs remain for further analysis.

### 2.3. Bioinformatic Analyses

To functionally annotate rs610261, we integrated data from HaploReg v4.2 and FIVEx databases. HaploReg leverages ENCODE and Roadmap Epigenomics data to predict potential regulatory roles of the variant on regulatory elements (e.g., enhancers, promoters), assess changes in transcription factor binding affinity, and evaluate evolutionary conservation [19]. Expression quantitative trait loci (eQTL) and splicing quantitative trait loci (sQTL) analyses were performed using FIVEx, which incorporates comprehensive genomic and transcriptomic datasets [20]. eQTL analysis employed linear regression models to associate the rs610261 genotype with *DENND2D* expression levels. Similarly, sQTL analysis used linear regression to assess the association between rs610261 and *DENND2D* transcript isoform abundance.

To assess the impact of *DENND2D* expression on prostate cancer outcomes, 40 public datasets from PCaDB [21], Gene Expression Database of Normal and Tumor Tissues 2 [22], and The Cancer Genome Atlas (TCGA) were examined. The TCGA Prostate Adenocarcinoma (PRAD) data were also used to explore *DENND2D*-related molecular mechanisms via gene ontology and hallmark pathway enrichment analyses using LinkedOmics [23]. Gene set enrichment analysis (GSEA) was conducted using LinkedOmics to identify gene sets correlated with *DENND2D* expression. Genes were ranked based on Pearson correlation coefficients between their expression levels and *DENND2D* expression. GSEA employs a weighted Kolmogorov–Smirnov-like running sum statistic [24] to calculate the Enrichment Score (ES), which quantifies the overrepresentation of gene set members at the extremes of the ranked gene list. This weighting scheme prioritizes genes at the top and bottom of the ranked list by incorporating gene rank into the running sum calculation. The ES represents the maximum deviation from zero encountered during this process. Statistical significance was determined by 1000 gene set label permutations, and multiple testing correction was performed using the Benjamini–Hochberg method to control the false discovery rate (FDR). Prognostic assessments of *DENND2D* and *MYC* target genes, such as mitotic arrest deficient 2 like 1 (*MAD2L1*), enhancer of rudimentary homolog mRNA splicing and mitosis factor (*ERH*), and chloride nucleotide-sensitive channel 1A (*CLNS1A*), were conducted using the TCGA-PRAD dataset.

*DENND2D* copy number variations were analyzed using Genomic Identification of Significant Targets In Cancer (GISTIC) 2.0 [25] applied to TCGA PRAD segmented copy number data. GISTIC values were used to define copy number categories: -2 (deep deletion), -1 (arm-level deletion), 0 (diploid), 1 (arm-level gain), and ≥2 (high amplification). Tumor-infiltrating immune cell levels were assessed in relation to *DENND2D* alterations and expression using the Tumor Immune Estimation Resource (TIMER) [26]. TIMER employs a statistical deconvolution method to estimate immune cell infiltration based on gene expression data from TCGA. This method uses pre-defined gene sets specific to various immune cell types (B cells, CD4^+^ T cells, CD8^+^ T cells, macrophages, neutrophils, and dendritic cells) and infers their relative abundance within the tumor microenvironment.

### 2.4. Statistical Analyses

Kaplan–Meier and log-rank tests were used to evaluate survival differences across genotypes or gene expression groups. Cox regression analyses, both univariate and multivariate, were used to calculate hazard ratios (HRs) and 95% confidence intervals (CIs) to analyze the associations between clinical characteristics and patient prognosis. The relationships between *DENND2D* expression and tumor characteristics were determined using Pearson’s and Spearman’s correlations. Statistical analyses were conducted using SPSS v19.0.0 (IBM, Armonk, NY, USA), with a two-sided *p*-value < 0.05 being considered statistically significant. Additionally, *DENND2D* expression in prostate cancer and normal tissues was compared using standardized mean differences (SMD) and 95% CIs. Associations between *DENND2D* mRNA levels and survival outcomes were evaluated by pooling the HRs and CIs in a random-effects model using Review Manager v5.4.1 (Cochrane, London, UK).

## 3. Results

**Figure 1:**
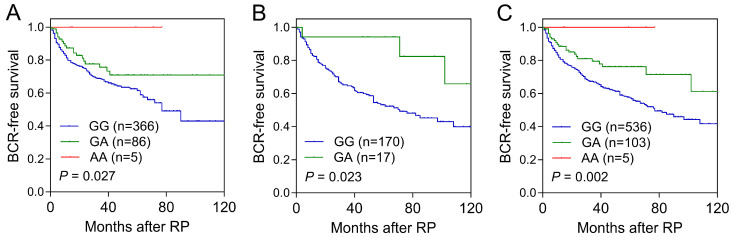
Kaplan–Meier curves show a significant association between *DENND2D* rs610261 and biochemical recurrence (BCR)-free survival in the (**A**) discovery, (**B**) replication, and (**C**) combined cohorts. RP, radical prostatectomy.

We conducted a Cox regression analysis to investigate the association between genetic variants in the *DENN* family and the risk of BCR (Table S2). Among the 151 SNPs analyzed in the discovery cohort, three SNPs within *DENND1A*, one in *DENND1C*, two in *DENND2A*, two in *DENND2D*, two in *DENND3*, one in *DENND6B*, and one in *DENND11* were found to be associated with BCR-free survival (*p* < 0.05). Notably, *DENND2D* rs610261 was the only SNP that was identified as consistently significant in the replication cohort (*p* = 0.032; Table 1 and Figure 1B). In a combined analysis, the *DENND2D* rs610261 G>A variant was associated with the significantly improved BCR-free survival. This finding was supported by the results of the multivariable Cox regression analyses adjusted for clinical factors such as age, PSA levels at diagnosis, pathological stage, Gleason score, surgical margin status, and lymph node metastasis (adjusted HR = 0.39, 95% CI = 0.23–0.66, *p* = 0.001; Table 1 and Figure 1C).

**Figure 2:**
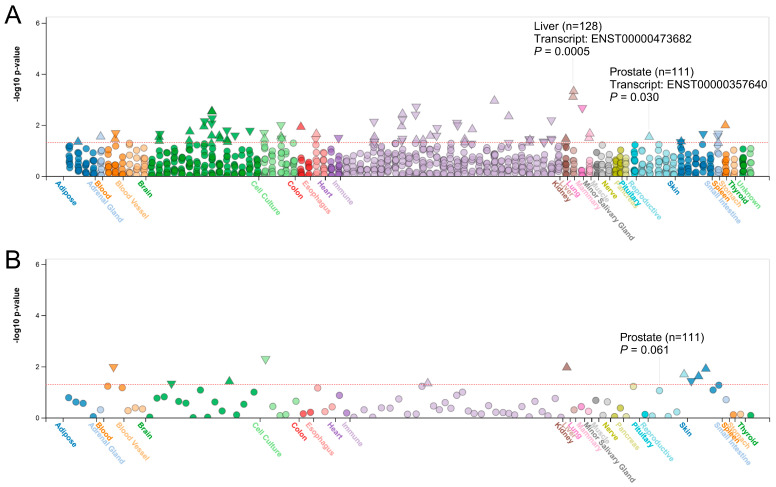
Functional implications of *DENND2D* rs610261: (**A**) Splicing quantitative trait loci and (**B**) expression quantitative trait loci analyses displaying the significant associations between rs610261 and *DENND2D* transcripts in multiple human tissues. The red horizontal line represents the nominal significance threshold (*p* = 0.05). Triangles indicate a positive effect of rs610261 on *DENND2D* expression, inverted triangles indicate a negative effect, and circles represent no significant effect.

To investigate the functional significance of rs610261, we utilized the HaploReg database, which indicated that this SNP resides within regions enriched for promoter/enhancer histone marks and DNase I hypersensitivity sites across various tissues, highlighting its potential regulatory role (Table S3). Findings from the FIVEx database further supported this hypothesis by showing that the protective A allele of rs610261 was linked to the elevated expression of the *DENND2D* transcript ENST00000357640 in prostate (*p* = 0.030, Figure 2A) and in other human tissues, with the strongest association observed for the *DENND2D* transcript ENST00000473682 in the liver (*p* = 0.0005). In eQTL analysis, the A allele only showed a suggestive increase in *DENND2D* expression in the prostate (*p* = 0.061), though significant associations were observed in multiple tissues, including the brain, immune cells, kidneys, reproductive organs, and skin (Figure 2B). These findings suggest that splicing in *DENND2D* may play an important role in the tissue-specific regulation mediated by rs610261.

**Figure 3:**
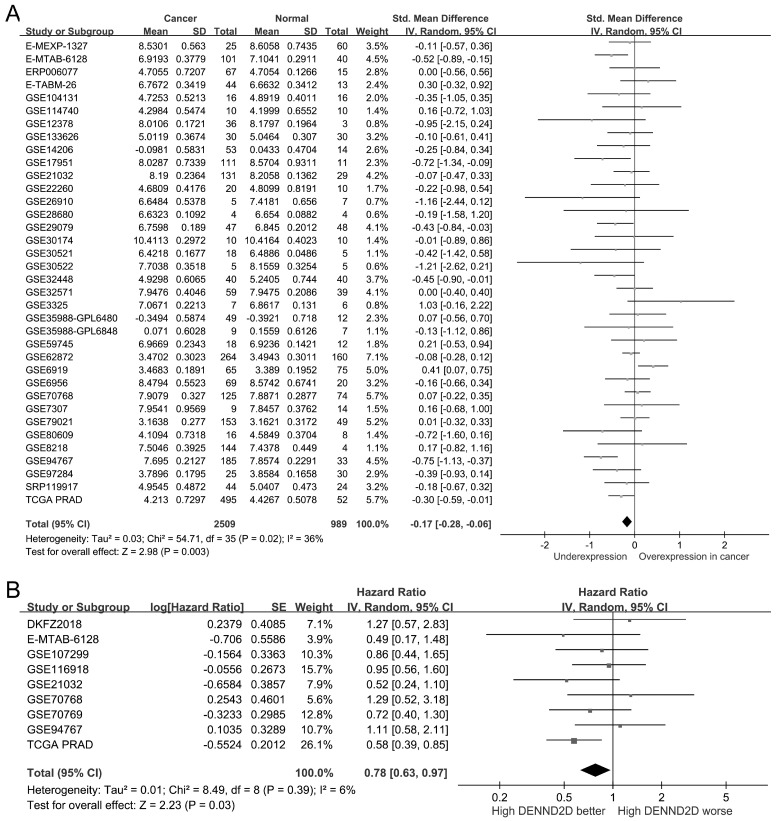
Clinical significance of *DENND2D* expression in prostate cancer: (**A**) *DENND2D* expression was significantly lower in prostate cancer tissues than in normal tissues using the analysis of 36 public datasets. (**B**) Pooled analysis revealed that higher *DENND2D* expression was associated with improved overall survival in patients with prostate cancer. Samples were stratified into low- and high-expression groups based on median expression values. SD, standard deviation. SE, standard error. IV, inverse variance. CI, confidence interval. Std, standardized. TCGA PRAD, The Cancer Genome Atlas Prostate Adenocarcinoma. df, degrees of freedom.

To evaluate the clinical relevance of *DENND2D* expression in prostate cancer, we analyzed the data from 2509 prostate cancer samples and 989 normal prostate tissue samples from 36 public datasets. *DENND2D* expression was significantly lower in prostate cancer tissues than in normal tissues (SMD = −0.17, 95% CI = −0.28 to −0.06, *p* = 0.003; Figure 3A). Furthermore, a pooled analysis of nine studies indicated that higher *DENND2D* expression correlated with improved prognosis (HR = 0.78, 95% CI = 0.63–0.97, *p* = 0.03; Figure 3B), suggesting a potential tumor-suppressive role for *DENND2D* in prostate cancer progression.

**Figure 4:**
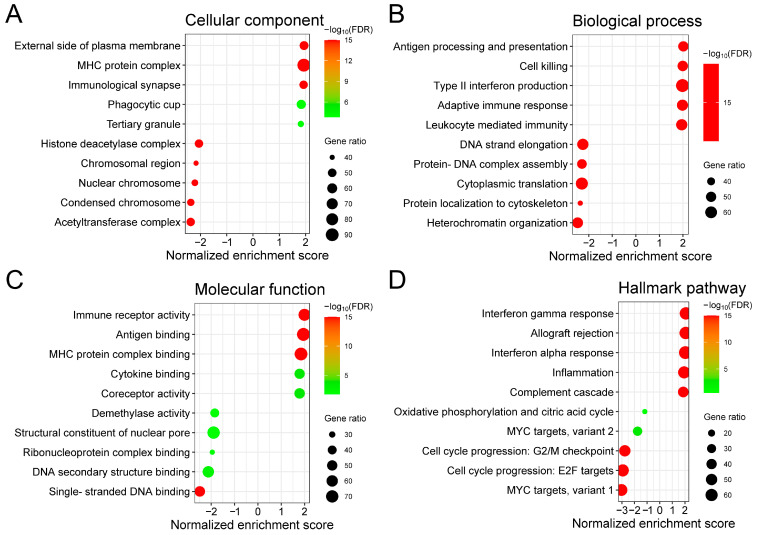
Gene Ontology and pathway analyses of genes correlated with *DENND2D* expression. Top 10 Gene Ontology terms in (**A**) cellular components, (**B**) biological processes, and (**C**) molecular functions. (**D**) Top 10 enriched Hallmark pathways. The size of the bubble indicates the number of enriched genes and color indicates the significance of the enrichment.

To investigate the biological role of *DENND2D* in prostate cancer, we identified genes that show correlated expression with *DENND2D* in the TCGA-PRAD dataset. In total, 4610 genes were positively correlated and 2353 genes were negatively correlated with *DENND2D*, with an FDR of <0.01 (Pearson’s correlation). GSEA was performed using the complete ranked list of genes based on correlation coefficients and *p*-values. Gene Ontology analysis revealed that the negatively correlated genes were enriched in cellular components, such as the acetyltransferase complex, condensed chromosomes, and nuclear chromosomes (Figure 4A). Additionally, they were involved in biological processes, such as heterochromatin organization, protein localization to the cytoskeleton, and cytoplasmic translation (Figure 4B), as well as molecular functions, such as single-stranded DNA binding and ribonucleoprotein complex binding (Figure 4C). Hallmark pathway analysis showed enrichment of pathways related to the negative regulation of *MYC* target genes and cell cycle progression (Figure 4D).

**Figure 5:**
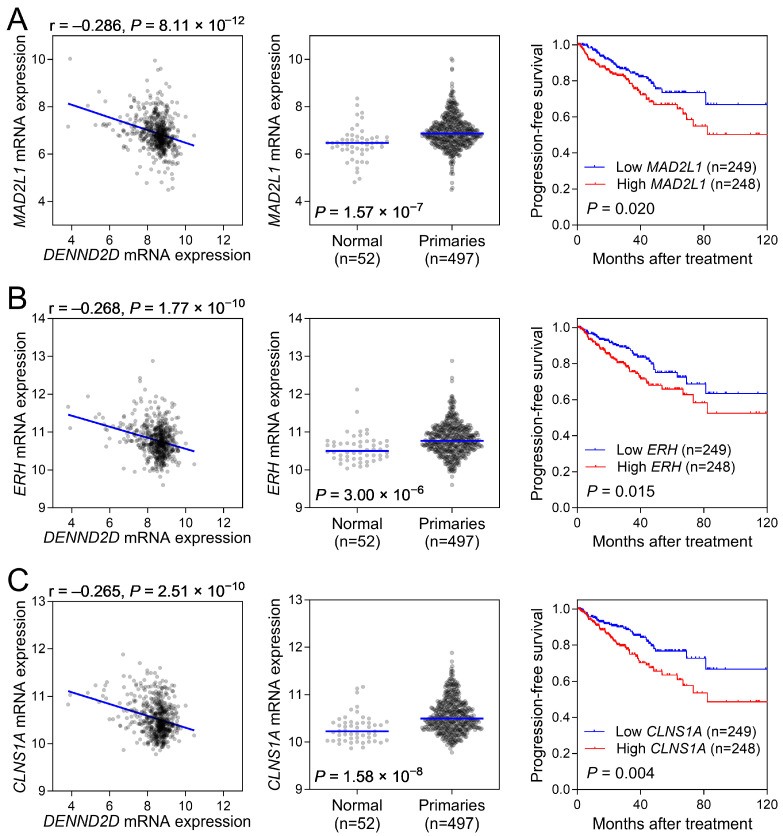
Association between *DENND2D* and *MYC* target gene expression in prostate cancer. The left panel illustrates the negative correlation between *DENND2D* and *MYC* target gene expression, including (**A**) *MAD2L1*, (**B**) *ERH*, and (**C**) *CLNS1A*, in prostate cancer. The middle panel shows increased expression of these *MYC* target genes in prostate cancer tissues compared to normal tissues. The right panel shows that the higher expression levels of *MAD2L1*, *ERH*, and *CLNS1A* are associated with worse survival outcomes in patients with prostate cancer. Expression data were log2(x + 1) transformed RNA-Seq by Expectation-Maximization normalized count. Samples were stratified into low- and high-expression groups based on median expression values.

GSEA identified “*MYC* targets v1” as the most significantly enriched pathway (normalized ES = −3.0371, FDR < 2.2 × 10^−16^), suggesting a link between *DENND2D* and *MYC* target genes. To validate this connection, we evaluated the correlation between *DENND2D* expression and the expression of genes within this highly enriched pathway. We discovered that *DENND2D* expression was inversely correlated with the expression of *MYC* target genes *MAD2L1*, *ERH*, and *CLNS1A* (Figure 5, left). Analysis of gene expression in prostate cancer tissues vs. normal tissues in the TCGA-PRAD dataset revealed significantly higher gene expressions in the prostate cancer tissues (Figure 5, middle). Moreover, their elevated expression levels were associated with poor survival outcomes, supporting *DENND2D*’s potential protective role through the suppression of *MYC* target genes in prostate cancer progression.

**Figure 6:**
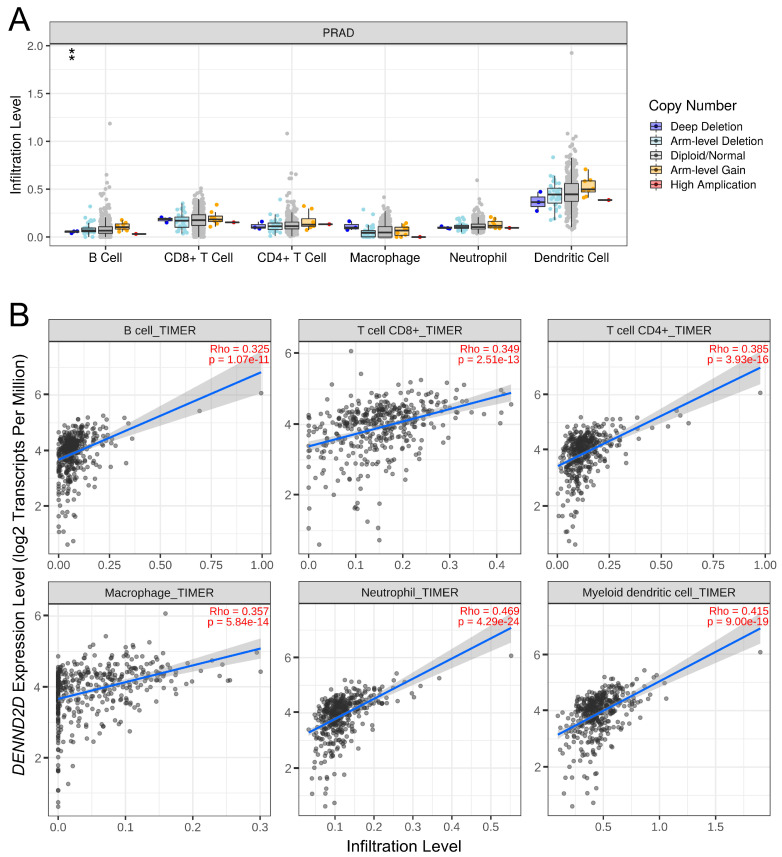
*DENND2D* expression and immune cell infiltration in prostate cancer: (**A**) The deep deletion of *DENND2D* correlated with reduced B-cell infiltration. The infiltration levels for each copy number category (deep deletion, n = 3; arm-level deletion, n = 34; diploid, n = 446; arm-level gain, n = 8; high amplification, n = 1) were compared to that of normal samples using a two-sided Wilcoxon rank-sum test. ** *p* <0.01. (**B**) Higher *DENND2D* expression positively correlated with the infiltration of B cells, CD8⁺ T cells, CD4⁺ T cells, macrophages, neutrophils, and dendritic cells.

As *DENND2D* was also implicated in the activation of interferon and inflammatory pathways in the enrichment analysis, we explored its relationship with immune cell infiltration in prostate cancer. Additionally, the deep deletion of *DENND2D* was linked to the reduced B cell infiltration (Figure 6A). Conversely, higher *DENND2D* expression positively correlated with the infiltration of various immune cells, including B cells, CD8^+^ T cells, CD4^+^ T cells, macrophages, neutrophils, and dendritic cells (Figure 6B). Thus, *DENND2D* is essential to the regulation of immune cell infiltration in prostate cancer.

## 4. Discussion

This study identified *DENND2D* and its genetic variant rs610261 as significant contributors to BCR risk in prostate cancer. The rs610261 A allele was associated with improved BCR-free survival, likely mediated by enhanced *DENND2D* expression. Functional analyses revealed that *DENND2D* exerts tumor-suppressive effects, with reduced *DENND2D* expression correlating with prostate cancer progression and poor prognosis. In addition, *DENND2D* expression was inversely associated with the expression of *MYC* target genes and showed a positive correlation with immune cell infiltration. Thus, *DENND2D* is a promising prognostic biomarker and target for therapeutic intervention in prostate cancer.

SNP rs610261 is located within an intronic region characterized by promoter- and enhancer-like chromatin modification patterns along with overlapping DNase I-hypersensitive sites, suggesting a potential regulatory role in *DENND2D* expression. *DENND2D* was determined to exert significant tumor-suppressive effects by inhibiting tumor progression in various cancers [12,27,28]. *DENND2D* downregulation or silencing, frequently driven by promoter hypermethylation, has been implicated in the development of non-small cell lung cancer (NSCLC) [29], esophageal squamous cell carcinoma [30], hepatocellular carcinoma [8], and gastric cancer [10]. Promoter hypermethylation of *DENND2D* correlates with the advanced disease stage, early recurrence, and poor prognosis. Demethylation-based reactivation of *DENND2D* expression restores its tumor-suppressive function, positioning *DENND2D* as a potential biomarker for aggressive cancers [8,30]. Studies indicate that *DENND2D* inhibits tumor proliferation and metastasis primarily by inducing apoptosis and suppressing survival pathways in tumor cells. The overexpression of *DENND2D* in NSCLC significantly reduced proliferation and tumorigenicity by promoting apoptosis [29]. Notably, *DENND2D* has also been identified as a direct target of miR-522, linking its regulation to microRNA-mediated control of tumor proliferation and metastasis in NSCLC. This accentuates a novel axis for therapeutic intervention [31]. Herein, the clinical relevance and biological functions of *DENND2D* in prostate cancer were examined. Analysis of the pooled datasets revealed that *DENND2D* expression was significantly reduced in prostate tumors, and patients with elevated *DENND2D* levels demonstrated improved survival. The expression networks associated with *DENND2D* were analyzed using GSEA, revealing an inverse relationship between the expression of *MYC*-regulated genes, including *MAD2L1*, *ERH*, and *CLNS1A*. *MYC* is a proto-oncogene encoding a transcription factor that regulates the expression of numerous genes involved in cell growth, proliferation, metabolism, and apoptosis. Aberrant *MYC* expression, commonly through gene amplification, translocation, or increased transcription, drives tumorigenesis by promoting uncontrolled cell proliferation and survival. Given that *MYC* expression was inversely correlated with *DENND2D* expression (Pearson’s r = −0.127, *p* = 0.005), the enrichment of *MYC* target pathways is expected. *MAD2L1* is a critical component of the spindle assembly checkpoint that ensures accurate chromosomal segregation during mitosis. *MAD2L1* dysregulation promotes tumorigenesis by inducing chromosomal instability and aneuploidy, and its overexpression has been linked to enhanced progression in various cancers [32]. Studies have demonstrated that *ERH,* a nuclear protein involved in cell cycle regulation and DNA replication, is frequently overexpressed in multiple cancer types wherein it modulates cell cycle checkpoints through interactions with mitotic kinases, thereby supporting the proliferation of rapidly dividing tumor cells [33]. *CLNS1A*, a chloride current regulator associated with the plasma membrane, interacts with various proteins to mediate diverse cellular functions, including the regulation of small nuclear ribonucleoprotein biosynthesis and cytoskeletal organization [34]. Additionally, *CLNS1A* amplification has been observed in malignant gliomas and breast cancer [35,36]. Notably, *CLNS1A* cooperates with the protein arginine methyltransferase 5, which functions as an epigenetic activator of androgen receptor transcription in castration-resistant prostate cancer [37]. Furthermore, the elevated expression of *DENND2D* was associated with the enhanced infiltration of immune cells, suggesting that *DENND2D* contributes to prostate cancer suppression through its combined effects on oncogenic pathways and immune modulation.

The advent of genomic tests has significantly enhanced the precision of prognostic tools in prostate cancer. By leveraging a 22-gene panel, Decipher has consistently demonstrated its ability to predict biochemical recurrence, metastasis, and cancer-specific mortality across multiple studies [38,39]. Although Decipher is a powerful classifier, its focus on a predefined gene set may overlook other relevant biomarkers or mechanisms involved in prostate cancer recurrence. *DENND2D* provides complementary insights into the molecular aggressiveness of prostate tumors, offering opportunities for refining treatment decisions and exploring novel therapeutic avenues. Nonetheless, further research is needed to elucidate the precise molecular mechanisms that underpin *DENND2D*’s tumor-suppressive functions in prostate cancer.

The strength of our study lies in its robust design, which combined genetic and functional analyses to comprehensively explore the role of *DENND2D* in prostate cancer. By integrating findings from discovery and replication cohorts, we ameliorated the validity of the association between rs610261 and improved BCR-free survival. Additionally, leveraging multiple datasets and conducting GSEA provided valuable insights into potential associations between *DENND2D* expression and tumor-suppressive mechanisms, including the regulation of *MYC* target genes and immune cell infiltration. This study has several limitations. The relatively small sample size reduced the statistical power of our results, particularly for less common genetic variants, and the exclusive inclusion of Taiwanese participants limited the generalizability of our data across ethnicities. Furthermore, the dependence on bioinformatic analyses for the functional interpretation of rs610261 necessitates experimental validation. The relatively short follow-up duration and focus on haplotype-tagged SNPs have restricted broader clinical implications. Although our findings reveal compelling associations between *DENND2D* and oncogenic processes, including *MYC* target gene regulation, cell cycle progression, and immune cell infiltration, our correlative analyses preclude definitive conclusions about causality. Future studies that address these limitations should strengthen our findings and their applicability.

## 5. Conclusions

In summary, this study highlights the clinical and biological significance of *DENND2D* in prostate cancer. Using a robust discovery–replication design, we provided compelling evidence that the *DENND2D* rs610261 G>A variant is a promising prognostic biomarker for prostate cancer recurrence. Moreover, the tumor-suppressive effects of *DENND2D*, which are mediated through *MYC* pathway regulation and immune cell infiltration, underscores its dual role in cancer control and immune modulation. While these findings advance our understanding of the role of *DENND2D* in prostate cancer, further research is needed to validate these results in larger cohorts and explore the therapeutic potential of targeting *DENND2D* in clinical settings.

## Figures and Tables

**Table 1:** Association of *DENND2D* rs610261 with biochemical recurrence after radical prostatectomy.

Genotype	Discovery				Replication				Combined			
	Patients	BCR	HR (95% CI)	*p*	Patients	BCR	HR (95% CI)	*p*	HR (95% CI)	*p*	HR (95% CI) ^a^	*p* ^a^
GG	366	118	1.00		170	88	1.00		1.00		1.00	
GA	86	19	0.66 (0.40–1.07)	0.089	17	4	0.33 (0.12–0.91)	0.032	0.56 (0.36–0.86)	0.008	0.42 (0.24–0.72)	0.002
AA	5	0	-	-	0	0	-	-	-	-	-	-
Trend			0.60 (0.38–0.95)	0.029			0.33 (0.12–0.91)	0.032	0.52 (0.34–0.80)	0.002	0.39 (0.23–0.66)	0.001

Abbreviations: BCR, biochemical recurrence; HR, hazard ratio; CI, confidence interval. ^a^ Adjustment for age, prostate-specific antigen at diagnosis, pathologic stage, Gleason score, surgical margin, and lymph node metastasis. -, Not estimated due to no event.

## Data Availability

Data will be available on reasonable request.

## Supplementary Materials

The following supporting information can be downloaded at: https://www.mdpi.com/article/10.3390/biomedicines13010025/s1. Table S1. Clinicopathologic characteristics of the study populations. Table S2. Association between *DENN* domain-containing gene polymorphisms and biochemical recurrence after radical prostatectomy. Table S3. Regulatory annotation of *DENND2D* rs610261.
