# Supplementary material for: Unveiling DENND2D as a Novel Prognostic Biomarker for Prostate Cancer Recurrence: From Gene to Prognosis
Source: Biomedicines. 2024 Dec 26;13(1):25. doi: 10.3390/biomedicines13010025 (PMC11760481; doi:10.3390/biomedicines13010025)
Supplement: Supplementary file 1 [file biomedicines-13-00025-s001.zip › biomedicines-3369172-supplementary.pdf]

Table S1. Clinicopathologic characteristics of the study populations

Characteristic	Discovery (n = 457)	Replication (n = 187)
Age at diagnosis		
Median, years (IQR)	66 (62-70)	66 (61-70)
PSA at diagnosis		
Median, ng/ml (IQR)	10.9 (6.9-18.4)	10.8 (7.1-17.6)
Pathologic stage, n (%)		
T1/T2	235 (51.4)	129 (70.9)
T3/T4/N1	222 (48.6)	53 (29.1)
Pathologic Gleason score, n (%)		
2-7	371 (81.2)	160 (85.6)
8-10	86 (18.8)	27 (14.4)
Surgical margin, n (%)		
Negative	307 (67.2)	153 (81.8)
Positive	150 (32.8)	34 (18.2)
Lymph node metastasis, n (%)		
Negative	325 (94.5)	180 (97.8)
Positive	19 (5.5)	4 (2.2)
Biochemical recurrence, n (%)	137 (30.0)	92 (49.2)
Median follow-up, months	38	74

Abbreviations: IQR, interquartile range; PSA, prostate-specific antigen.

Subtotals do not sum to n of patients due to missing data.

Table S2. Association between *DENN* domain containing gene polymorphisms and biochemical recurrence after radical prostatectomy

Gene	SNP ID	Chromosome	Position	Allele	Genotype[a]	MAF	HWE	*P*
DENND10	rs75277104	10	119104196	C>G	0/47/402	0.052	0.623	0.691
DENND10	rs3858336	10	119118198	T>C	90/215/148	0.437	0.448	0.223
DENND10	rs3802739	10	119129510	T>C	85/223/147	0.431	0.924	0.604
DENND11	rs2269984	7	141662893	T>A	104/224/129	0.472	0.780	0.903
DENND11	rs17162413	7	141673106	G>A	37/202/218	0.302	0.320	0.026
DENND1A	rs4836922	9	123391489	A>C	17/134/304	0.184	0.642	0.890
DENND1A	rs2808413	9	123393271	A>C	64/215/177	0.378	0.921	0.628
DENND1A	rs76835710	9	123401621	A>G	21/138/297	0.197	0.375	0.924
DENND1A	rs28647805	9	123406270	A>G	1/81/374	0.091	0.157	0.693
DENND1A	rs67595214	9	123407370	G>A	20/157/279	0.214	0.890	0.832
DENND1A	rs7048861	9	123415581	A>G	70/221/165	0.398	0.771	0.825
DENND1A	rs12351628	9	123415868	T>C	32/199/224	0.288	0.210	0.892
DENND1A	rs7851052	9	123416925	C>T	66/205/184	0.369	0.485	0.321
DENND1A	rs144437107	9	123420349	C>G	1/49/406	0.055	1.000	0.273
DENND1A	rs73664359	9	123424504	G>A	5/63/389	0.079	0.190	0.162
DENND1A	rs12335741	9	123426360	G>A	2/61/392	0.072	1.000	0.116
DENND1A	rs76274475	9	123435079	G>A	4/62/390	0.076	0.321	0.908
DENND1A	rs4568669	9	123437716	C>T	47/191/216	0.313	0.664	0.146
DENND1A	rs3829851	9	123457427	A>G	0/54/401	0.060	0.394	0.273
DENND1A	rs10760289	9	123465583	C>A	46/199/212	0.318	1.000	0.211
DENND1A	rs59460368	9	123472792	A>G	12/122/319	0.161	1.000	0.323
DENND1A	rs77086544	9	123486158	C>A	7/88/360	0.112	0.489	0.471
DENND1A	rs2564365	9	123487789	A>C	29/171/256	0.251	1.000	0.198

DENND1A	rs2772208	9	123500653	A>G	37/185/234	0.284	1.000	0.131
DENND1A	rs116868115	9	123509572	G>A	0/54/402	0.059	0.390	0.010
DENND1A	rs2772215	9	123520720	A>C	11/97/346	0.131	0.215	0.089
DENND1A	rs10818827	9	123524416	G>A	15/160/281	0.209	0.203	0.153
DENND1A	rs1888027	9	123541092	G>A	27/176/253	0.253	0.622	0.027
DENND1A	rs4838066	9	123542027	T>C	1/61/393	0.069	0.712	0.704
DENND1A	rs7027071	9	123542763	G>A	1/51/402	0.060	1.000	0.257
DENND1A	rs75940099	9	123544358	C>T	2/42/412	0.050	0.312	0.281
DENND1A	rs10986049	9	123555062	G>A	4/67/385	0.083	0.537	0.089
DENND1A	rs7855233	9	123566048	A>G	30/184/242	0.267	0.553	0.034
DENND1A	rs7020626	9	123576203	G>A	30/190/234	0.275	0.348	0.070
DENND1A	rs7857782	9	123579031	A>G	16/155/285	0.204	0.473	0.114
DENND1A	rs7852296	9	123589939	G>A	5/59/392	0.076	0.169	0.289
DENND1A	rs4836937	9	123598049	A>G	19/167/265	0.226	0.286	0.254
DENND1A	rs77991954	9	123599492	G>A	3/61/392	0.075	0.733	0.740
DENND1A	rs7855648	9	123618160	A>G	20/184/250	0.247	0.059	0.069
DENND1A	rs913783	9	123671824	A>G	15/168/272	0.218	0.075	0.428
DENND1A	rs7028815	9	123673090	A>G	4/86/367	0.103	0.805	0.268
DENND1A	rs10818854	9	123684499	G>A	1/46/410	0.053	1.000	0.193
DENND1A	rs7860055	9	123700428	T>G	8/116/332	0.145	0.706	0.606
DENND1A	rs74909824	9	123703661	G>A	12/88/354	0.123	0.048	0.251
DENND1A	rs60775187	9	123710426	A>G	1/87/366	0.097	0.105	0.785
DENND1A	rs117913918	9	123712641	G>C	1/56/400	0.063	1.000	0.783
DENND1A	rs9785285	9	123757789	T>C	4/98/354	0.116	0.493	0.406
DENND1A	rs2479106	9	123762933	A>G	9/133/313	0.166	0.309	0.274

DENND1A	rs1752156	9	123772233	T>C	0/98/350	0.110	0.003	0.242
DENND1A	rs2799465	9	123782330	T>C	3/93/360	0.109	0.336	0.141
DENND1A	rs10986109	9	123799857	C>T	8/101/348	0.128	0.834	0.313
DENND1A	rs543070	9	123834268	A>G	1/78/375	0.087	0.235	0.755
DENND1A	rs142761957	9	123862994	T>C	2/42/410	0.050	0.314	0.344
DENND1A	rs143566847	9	123870762	G>A	2/52/399	0.062	0.691	0.055
DENND1B	rs2287537	1	197513135	G>A	52/210/194	0.344	0.680	0.279
DENND1B	rs4915551	1	197539771	A>G	9/105/343	0.135	0.841	0.053
DENND1B	rs16841754	1	197584103	T>C	58/185/210	0.334	0.092	0.733
DENND1B	rs1833464	1	197602447	A>G	9/100/347	0.129	0.537	0.105
DENND1B	rs10737693	1	197629900	C>T	23/152/282	0.216	0.680	0.805
DENND1B	rs2488389	1	197662011	G>A	22/137/275	0.208	0.383	0.995
DENND1B	rs12031377	1	197724136	C>T	104/211/134	0.467	0.257	0.782
DENND1B	rs2488397	1	197732149	G>C	21/157/278	0.217	1.000	0.941
DENND1B	rs17641524	1	197735587	C>T	5/75/376	0.092	0.573	0.406
DENND1B	rs10922273	1	197756243	C>T	117/216/121	0.496	0.305	0.701
DENND1B	rs1998598	1	197758512	A>G	22/159/271	0.224	0.893	0.798
DENND1C	rs117140126	19	6473005	G>A	2/46/403	0.056	0.643	0.017
DENND1C	rs3745571	19	6475602	T>C	67/193/193	0.359	0.104	0.568
DENND1C	rs149014244	19	6476197	G>A	2/42/412	0.051	0.332	0.613
DENND1C	rs3844450	19	6481448	A>C	108/233/113	0.495	0.640	0.481
DENND2A	rs74329726	7	140527023	G>T	0/48/409	0.052	0.625	0.499
DENND2A	rs6943131	7	140527673	G>A	5/130/320	0.155	0.032	0.088
DENND2A	rs4726889	7	140532443	A>G	18/114/316	0.166	0.062	0.605
DENND2A	rs7806065	7	140533407	T>G	5/110/338	0.134	0.309	0.392

DENND2A	rs16882335	7	140538206	T>C	0/68/385	0.074	0.160	0.121
DENND2A	rs10254745	7	140538407	G>A	5/110/338	0.134	0.309	0.082
DENND2A	rs4458784	7	140539505	A>G	5/117/335	0.140	0.172	0.236
DENND2A	rs4236489	7	140540555	G>A	96/247/113	0.480	0.094	0.254
DENND2A	rs2293176	7	140544827	G>A	3/109/345	0.127	0.089	0.115
DENND2A	rs1879901	7	140547094	G>A	3/102/352	0.119	0.179	0.109
DENND2A	rs12540766	7	140547259	T>C	2/107/343	0.124	0.030	0.050
DENND2A	rs669797	7	140564529	C>A	4/119/331	0.142	0.053	0.117
DENND2A	rs79659279	7	140578011	C>T	0/45/411	0.050	0.618	0.318
DENND2A	rs269241	7	140596601	C>T	11/119/326	0.158	0.860	0.233
DENND2A	rs78818827	7	140600566	T>C	21/155/280	0.215	1.000	0.725
DENND2A	rs269264	7	140630481	T>G	1/63/391	0.073	0.497	0.024
DENND2A	rs269263	7	140630533	C>T	14/104/334	0.148	0.064	0.043
DENND2A	rs10260676	7	140667353	C>T	6/83/366	0.107	0.334	0.417
DENND2B	rs72858217	11	8706412	C>T	1/46/409	0.052	1.000	0.978
DENND2B	rs10840114	11	8715148	T>C	51/226/180	0.358	0.128	0.597
DENND2B	rs75724455	11	8715201	T>G	2/80/372	0.092	0.408	0.830
DENND2B	rs4061048	11	8716203	A>G	24/143/286	0.210	0.263	0.226
DENND2B	rs56797102	11	8718274	A>G	26/133/293	0.204	0.044	0.169
DENND2B	rs3812762	11	8730093	G>C	24/125/306	0.190	0.032	0.159
DENND2B	rs11042055	11	8735309	G>A	40/227/189	0.337	0.012	0.713
DENND2B	rs3763920	11	8787040	G>A	44/213/200	0.328	0.290	0.891
DENND2B	rs12421789	11	8792409	A>G	101/236/115	0.484	0.348	0.230
DENND2B	rs11042078	11	8805153	G>T	33/226/197	0.321	0.003	0.828
DENND2B	rs79687170	11	8833639	G>A	2/73/382	0.084	0.758	0.227

DENND2B	rs2742552	11	8855305	T>C	97/214/144	0.448	0.346	0.708
DENND2B	rs1835790	11	8871751	C>A	0/95/354	0.105	0.009	0.770
DENND2B	rs2455601	11	8877853	G>A	35/169/253	0.261	0.278	0.557
DENND2B	rs2742540	11	8877981	A>G	95/216/145	0.445	0.451	0.874
DENND2C	rs12021674	1	114620202	C>T	56/218/182	0.359	0.545	0.493
DENND2D	rs3818797	1	111185988	T>C	63/221/173	0.379	0.621	0.220
DENND2D	rs2494015	1	111191500	T>C	59/218/179	0.369	0.548	0.781
DENND2D	rs657605	1	111194015	T>C	2/56/398	0.065	1.000	0.655
DENND2D	rs630505	1	111195294	T>C	2/58/394	0.068	1.000	0.596
DENND2D	rs17027070	1	111195556	C>T	7/91/356	0.116	0.648	0.816
DENND2D	rs610261	1	111198320	G>A	5/86/366	0.105	1.000	0.029
DENND2D	rs1807389	1	111202771	G>A	68/220/169	0.391	0.845	0.026
DENND2D	rs745527	1	111203353	A>G	7/115/334	0.140	0.562	0.255
DENND3	rs59107076	8	141120763	G>A	3/67/386	0.082	1.000	0.368
DENND3	rs57552733	8	141121729	C>T	17/149/291	0.201	0.772	0.189
DENND3	rs7839297	8	141121831	T>C	36/173/247	0.270	0.408	0.539
DENND3	rs307765	8	141142343	G>A	60/206/191	0.357	0.839	0.034
DENND3	rs307758	8	141147161	T>C	104/217/135	0.466	0.455	0.751
DENND3	rs3816063	8	141150965	C>A	11/140/305	0.177	0.338	0.004
DENND3	rs11782809	8	141167135	A>G	28/158/271	0.234	0.516	0.108
DENND3	rs118157870	8	141181158	C>T	3/53/401	0.065	0.428	0.513
DENND3	rs7001673	8	141188497	T>G	77/258/121	0.452	0.003	0.107
DENND4A	rs117746447	15	65674429	T>C	4/63/389	0.078	0.509	0.368
DENND4A	rs77965101	15	65707082	C>T	4/82/367	0.100	1.000	0.122
DENND4A	rs28687655	15	65715634	T>A	20/160/275	0.218	0.683	0.282

DENND4A	rs934542	15	65723838	G>A	1/45/409	0.051	1.000	0.187
DENND4A	rs6494537	15	65759007	T>C	16/138/300	0.186	1.000	0.262
DENND4A	rs2572207	15	65778355	T>C	18/156/280	0.210	0.672	0.325
DENND4A	rs2414884	15	65783534	T>C	16/138/303	0.184	0.878	0.210
DENND4B	rs1043608	1	153936593	G>C	39/173/244	0.276	0.352	0.411
DENND4C	rs7040288	9	19240006	A>G	18/172/265	0.228	0.145	0.960
DENND4C	rs1576385	9	19254451	C>T	23/149/284	0.214	0.408	0.561
DENND4C	rs10757048	9	19281101	A>G	97/239/120	0.475	0.305	0.903
DENND4C	rs118029367	9	19301937	A>G	1/59/396	0.067	0.711	0.091
DENND4C	rs61635462	9	19311858	C>A	2/64/389	0.075	1.000	0.710
DENND4C	rs2803122	9	19334017	C>T	85/239/133	0.448	0.258	0.902
DENND4C	rs7866416	9	19340523	A>G	24/149/283	0.216	0.337	0.629
DENND4C	rs76832559	9	19353899	T>G	2/53/402	0.063	0.698	0.654
DENND4C	rs12554577	9	19355549	G>T	38/193/223	0.296	0.911	0.399
DENND4C	rs147884803	9	19358892	C>T	0/60/390	0.066	0.245	0.517
DENND5A	rs117267653	11	9185724	G>A	0/50/405	0.056	0.385	0.510
DENND5A	rs35955530	11	9203069	G>A	112/224/114	0.498	0.925	0.954
DENND5A	rs34729888	11	9232258	T>C	115/224/117	0.498	0.710	0.977
DENND5B	rs2293038	12	31432679	G>T	43/187/227	0.299	0.579	0.536
DENND5B	rs1012154	12	31474650	T>C	7/99/351	0.123	1.000	0.726
DENND5B	rs10743759	12	31527316	A>G	9/120/327	0.151	0.717	0.748
DENND5B	rs4482118	12	31547112	T>C	10/118/326	0.152	1.000	0.783
DENND5B	rs1259359	12	31588139	G>T	5/95/357	0.114	0.819	0.461
DENND6A	rs8059	3	57625510	A>G	85/233/135	0.444	0.345	0.551
DENND6A	rs7627119	3	57659647	G>A	86/235/136	0.445	0.347	0.540

DENND6A	rs12629424	3	57688456	C>A	103/240/113	0.489	0.225	0.244
DENND6B	rs62241230	22	50316037	C>T	24/146/287	0.212	0.331	0.655
DENND6B	rs78477728	22	50319225	C>T	5/87/364	0.108	1.000	0.020

Abbreviations: SNP, single nucleotide polymorphism; MAF, minor alleles frequency; HWE, Hardy-Weinberg equilibrium.

[a] The number represents minor allele homozygotes/heterozygotes/major allele homozygotes.

Table S3. Regulatory annotation of *DENND2D* rs610261

Position	Allele (reference>alternate)	ASN frequency	Variant type	Promoter histone marks	Enhancer histone marks	DNAse	eQTL hits
111198320	G > A	0.15	Intronic	BLD, FAT, GI, MUS	BLD, GI, SPLN, LIV	PLCNT, BLD	1 hit

Abbreviations: ASN, Asian; eQTL, expression quantitative trait loci; BLD, blood; GI, gastrointestinal; MUS, muscle; SPLN, spleen; LIV, liver; PLCNT, Placenta.
